# Case report: A rare variant m.4135T>C in the *MT-ND1* gene leads to Leber hereditary optic neuropathy and altered respiratory chain supercomplexes

**DOI:** 10.3389/fgene.2023.1182288

**Published:** 2023-05-18

**Authors:** Tereza Rákosníková, Silvie Kelifová, Hana Štufková, Kateřina Lokvencová, Petra Lišková, Bohdan Kousal, Tomáš Honzík, Hana Hansíková, Václav Martínek, Markéta Tesařová

**Affiliations:** ^1^ Laboratory for Study of Mitochondrial Disorders, Department of Paediatrics and Inherited Metabolic Disorders, First Faculty of Medicine and General University Hospital in Prague, Charles University, Prague, Czechia; ^2^ Department of Ophthalmology, First Faculty of Medicine, Charles University and General University Hospital in Prague, Prague, Czechia; ^3^ Department of Biochemistry, Faculty of Science, Charles University, Prague, Czechia

**Keywords:** mitochondria, optic neuropathy, mtDNA, complex I, supercomplexes

## Abstract

Leber hereditary optic neuropathy is a primary mitochondrial disease characterized by acute visual loss due to the degeneration of retinal ganglion cells. In this study, we describe a patient carrying a rare missense heteroplasmic variant in *MT-ND1*, NC_012920.1:m.4135T>C (p.Tyr277His) manifesting with a typical bilateral painless decrease of the visual function, triggered by physical exercise or higher ambient temperature. Functional studies in muscle and fibroblasts show that amino acid substitution Tyr277 with His leads to only a negligibly decreased level of respiratory chain complex I (CI), but the formation of supercomplexes and the activity of the enzyme are disturbed noticeably. Our data indicate that although CI is successfully assembled in the patient’s mitochondria, its function is hampered by the m.4135T>C variant, probably by stabilizing CI in its inactive form. We conclude that the m.4135T>C variant together with a combination of external factors is necessary to manifest the phenotype.

## 1 Introduction

Leber hereditary optic neuropathy (LHON, MIM #535000) is the most frequent mitochondrial disease. It is characterized by progressive painless visual deterioration, usually resulting in irreversible blindness. In more than 90% of cases, LHON is caused by one of the common mitochondrial DNA (mtDNA) pathogenic variants (*MT-ND4* NC_012920.1:m.11778G>A, *MT-ND6* NC_012920.1:m.14484T>C, and *MT-ND1* NC_012920.1:m.3460G>A). Recently, biallelic variants in nuclear genes, particularly *DNAJC30*, were identified (arLHON, MIM #619382). Most LHON disease variants are found in complex I (CI)-related genes localized either in the mitochondrial or the nuclear genome (Maresca and Carelli, 2021). Apart from LHON, CI deficiency may manifest as multisystem syndromes, such as mitochondrial encephalomyopathy, lactic acidosis, and stroke-like episodes (MELAS) or Leigh syndrome.

Respiratory chain CI (NADH: coenzyme Q oxidoreductase, EC 1.6.5.3) is the largest enzyme of the oxidative phosphorylation system (OXPHOS) and consists of 44 different protein subunits. The mtDNA encodes seven subunits, and the nuclear genome encodes the rest. CI comprises three modules, namely, the N-module, where NADH is oxidized and electrons are passed through the iron–sulfur clusters to the Q-module, where ubiquinone is subsequently reduced, and the transmembrane part of the enzyme, the P-module, which pumps four protons for every two electrons. The mtDNA-encoded membrane core subunits (ND1—ND6 and ND4L) are localized in the P-module. The assembly of CI is an intricate process, in which numerous assembly intermediates are formed in several parallel steps to produce a fully assembled CI. Moreover, CI also forms supercomplexes (SCs) with a complex III (CIII)-dimer (I + III_2_) or with CIII-dimer and one or two copies of complex IV (CIV) (I+III_2_+IV_1-2_) (Calvo et al., 2020).

Herein, we present a patient with a unique manifestation of LHON due to a rare missense variant in *MT-ND1*, NC_012920.1:m.4135T>C. By analyzing CI activity and SC formation, we provide evidence for the pathogenicity of the variant.

## 2 Case description

The male patient was referred to our department at the age of 38 years for one-year-lasting symptoms of fluctuating declines of visual acuity bilaterally occurring only during physical exercise or higher ambient temperatures (i.e. Uhthoff phenomenon). He was not able to distinguish which eye was initially affected. His medical history was uneventful, including his ocular history. He has been a heavy smoker (15 cigarettes a day) since he was 15.

His family history revealed that his father suffers from color vision deficiency. One of his two older brothers died of a heart defect, not closely specified, at age 15 (no biological material was available for genetic testing); his mother and the other brother are healthy. The older brother is also a smoker but has been smoking since he was 18 and reported fewer cigarettes smoked per day (seven). His ophthalmic examination at 42 revealed no pathology besides color vision impairment, which was attributed to other genetic causes.

**FIGURE 1:**
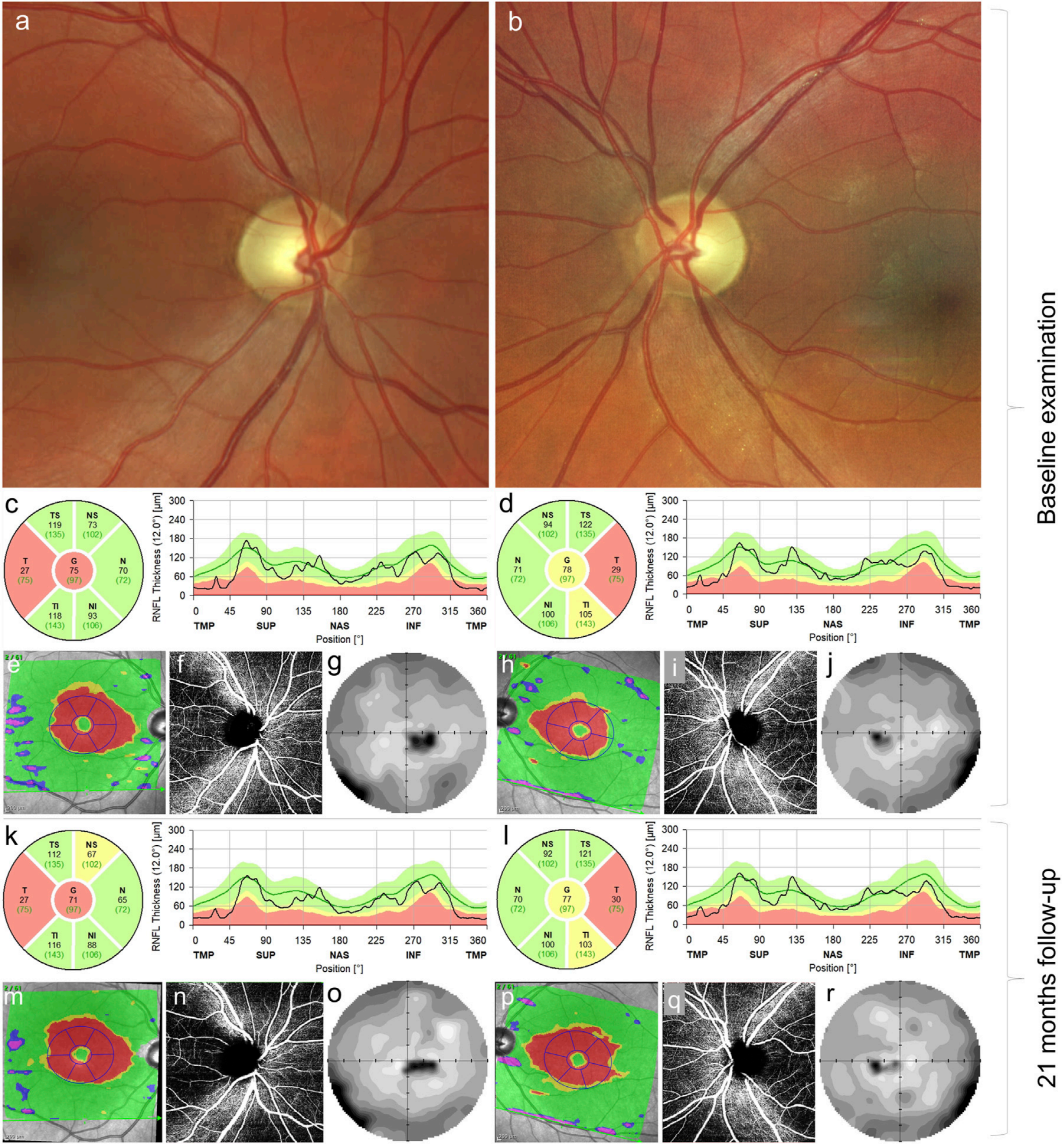
Ocular findings in the case carrying m.4135T>C. Fundus photograph of the right **(A)** and left eyes **(B)** documenting pallor (atrophy) of the optic discs. Thinning of the retinal nerve fiber layer in the right **(C)** and left **(D)** eyes in the temporal quadrants at baseline examination corresponding to thinning of the ganglion cell layer (red areas) **(E,H)** in the macular region, peripapillary capillary dropout **(F,I)** and centrocecal scotoma more pronounced on the right **(G)** than in the left **(J)** eye. Follow-up examination 21 months later, retinal nerve fiber layer in the right **(K)** and left **(L)** eyes, ganglion cell layer in the right **(M)** and left **(P)** eyes, bilateral peripapillary capillary dropout **(N, Q),** and visual field defect in the right **(O)** and left **(R)** eyes; no marked progression was observed.

An initial ocular examination performed 1 year after the onset of visual symptoms revealed best-corrected visual acuity (BCVA) 0.32 (0.50 logMAR) in the right eye and 0.5 (0.30 logMAR) in the left eye. Color vision assessment using the Lanthony desaturated D-15 test showed marked bilateral color vision impairment. Static perimetry (M700, Medmont International, Nunawading, Australia) showed bilateral centrocecal scotoma with corresponding retinal nerve fiber layer (RNFL) thinning, as measured by spectral-domain optical coherence tomography and decreased central vision. Fundus examination revealed pallor of the optic disc bilaterally (Figure 1). No other ocular or systemic pathology was found. Selective metabolic screening showed only mild intermittent hyperalaninaemia (maximum 591 μmol/L; controls 150—500 μmol/L), lactate concentration in blood and urine, and urine organic acids were repeatedly normal. A metabolic examination was also performed on the older brother with normal findings.

Because of a history of painless subacute onset of visual impairment, LHON was suspected. Therefore, idebenone treatment (900 mg/day) was started 1 year and 3 months after the onset. Even though known LHON pathogenic mutations were excluded, later genetic testing confirmed the *MT-ND1* variant, supporting our previous decision to start the treatment. Then, 21 months after the disease manifestation, BCVA was 0.25 (0.60 logMAR) and 0.5 (0.3 logMAR) in the right and left eyes, respectively. Visual field defects remained stable, as well as the reduction of RNFL in temporal fields bilaterally (Figure 1). The Uhthoff phenomenon persisted subjectively.

## 3 Material and methods

The study was approved by the Ethics Committee of the General University Hospital in Prague and was conducted in agreement with institutional guidelines. A written informed consent for participation in the study was obtained from the patient and his brother.

### 3.1 Mitochondrial DNA mutation analysis

Genomic DNA (gDNA) from all available samples was isolated according to the manufacturer’s instructions (QIAGEN, Hilden, Germany) using the following kits: the Gentra Puregene Blood Kit (blood samples), QIAamp DNA Micro Kit (buccal swabs and urine epithelial cells), and QIAamp DNA Mini Kit (muscle biopsy and fibroblasts).

A complete mtDNA genome sequence in blood, muscle, and fibroblast cells was analyzed using SeqCap EZ Design: Mitochondrial Genome Design (Roche NimbleGen, Pleasanton, CA, United States) enrichment kit, followed by analysis on the MiSeq (Illumina, San Diego, CA, United States) system. Cambridge Reference Sequence (NC_012920.1) was used for mtDNA variant annotation. The heteroplasmy was determined as the percentage of reads with the mutated variant. To determine heteroplasmy levels in buccal swabs and urine epithelial cells, the mtDNA region m.3803–m.4410 was amplified by PCR, followed by analysis on the MiSeq sequencer. No significant differences were found in the heteroplasmy levels between the methods tested in blood samples. The detection limit of both methods is 3%.

### 3.2 Exome sequencing

Exome sequencing was performed using gDNA extracted from the leukocytes of the proband. Exome enrichment was performed on individually barcoded samples using KAPA HyperExome Probes (Roche). NovaSeq 6000 platform (Illumina) with 100 bp paired-end reads was used for sequencing. Data processing and variant annotation proceeded as described previously (Neřoldová et al., 2016). Variants were filtered based on the population frequencies using a GnomAD v2.1.1 public database (Karczewski et al., 2020) and an in-house database of Czech population-specific variants from more than 2,100 alleles. Candidate variants were prioritized for autosomal dominant, autosomal recessive, and X-linked inheritance patterns. The functional impact and the conservation score were also considered. When reanalyzing data, specific attention was paid to optic neuropathy genes (v3.7 https://panelapp.genomicsengland.co.uk), including *TMEM126A*, previously reported with Uhthoff´s phenomenon (Désir et al., 2012), and CI-related genes.

### 3.3 Isolation of mitochondria

*Musculus triceps surae* biopsy was obtained in local anesthesia, and mitochondria were isolated as described previously (Danhelovska et al., 2020). Briefly, the samples were transported on ice (at 4°C) and mitochondria were isolated immediately by standard differential centrifugation in buffer containing 150 mM KCl, 50 mM Tris/HCl (pH 7.5), 2 mM EDTA, and 2 μg/mL aprotinin. Studied skin fibroblasts were cultivated under the standard condition in high-glucose Dulbecco’s modified Eagle medium (DMEM; Pan Biotech, Aidenbach, Germany) supplemented with 10% (v/v) fetal bovine serum (GE Healthcare, Chicago, IL, United States) and antibiotic–antimycotic solution (Biosera, Nuaile, France), as described previously (Daňhelovská et al., 2021), and did not exceed passage 10. For Blue Native Polyacrylamide Gel Electrophoresis (BN-PAGE), mitochondria were isolated by standard differential centrifugation (Stiburek et al., 2005).

### 3.4 Native electrophoresis and Western blotting

To analyze the steady-state levels of OXPHOS protein complexes, BN-PAGE of n-dodecyl-β-D-maltopyranoside (DDM)-solubilized isolated mitochondria was used (final ratio 4.8 or 6 mg DDM/mg muscle or fibroblasts protein, respectively). The protein concentration was determined by BCA assay (Thermo Fisher Scientific, Waltham, MA, United States). An amount of 10–20 μg of protein was loaded per lane and separated by 6–15% or 4–14% (w/v) polyacrylamide gradient gels (MinProtean^®^3 system; Bio-Rad, Hercules, CA, United States). A study of SCs was performed using BN-PAGE of digitonin (DIG)-solubilized isolated mitochondria (final ratio 7 mg DIG/mg protein). A total of 15 μg of protein was loaded per lane and separated by NativePAGE™ 3–12% Bis-Tris Mini Protein gels (Thermo Fisher Scientific). BN-PAGE gels were transferred onto the immobilon-P PVDF Membrane (Millipore, Burlington, MA, United States) by semi-dry electroblotting using the Hoefer semi-dry transfer unit (Hoefer, Harvard Bioscience, Holliston, MA, United States).

Primary detection of BN-PAGE blots was performed using mouse monoclonal antibodies against OXPHOS subunits (Abcam, Cambridge, United Kingdom). In fibroblasts, CI was detected by three different antibodies (NDUFA9, NDUFV1, and NDUFS3). The immunoblots were detected with peroxidase-conjugated secondary antibodies by chemiluminescence using G:Box (Syngene, Cambridge, United Kingdom) and analyzed by Quantity one (Bio-Rad) or ImageJ 1.48 v (Wayne Rasband, National Institutes of Health, Bethesda, Maryland, United States), as described previously (Daňhelovská et al., 2021).

### 3.5 Spectrophotometry

Respiratory chain complex (CI − NADH:coenzyme Q oxidoreductase; complex I + III − NADH:cytochrome *c* oxidoreductase; complex II − succinate-coenzyme Q oxidoreductase; complex II + III − succinate:cytochrome *c* oxidoreductase; complex III − coenzyme Q:cytochrome *c* oxidoreductase; and complex IV − cytochrome *c* oxidase) activities were measured according to Rustin et al. (1994), and citrate synthase (serving as the control enzyme to avoid assay variability) activity was measured according to Srere (1969). Protein concentrations were measured by the Lowry method (Lowry et al., 1951).

### 3.6 Computational structural analyses

The visualizations of respiratory chain complexes and their components were rendered by PyMol software, using atomic coordinates of human CI (PDB ID: 5XTD) and coordinates of active and inactive forms of mouse CI (PDB ID: 6G2J and 6G72, respectively) (Guo et al., 2017). The effect of mutations on protein structure and stability was predicted using DynaMut software (Rodrigues et al., 2018). DynaMut integrates their graph-based signatures along with normal-mode dynamics to generate a consensus prediction of the impact of a variant on protein stability, thus allowing prediction of both stabilizing and destabilizing effects of the missense variant on the protein.

Multiple sequence alignment was performed using the ConSurf server (Ashkenazy et al., 2016). The resulting alignment contains 2,000 unique sequences sharing identity between 50% and 95% with the human ND1 that sampled the representative homologous sequences in equal intervals (Supplementary file S1 Data Sheet 1.zip).

## 4 Results

### 4.1 Molecular genetic analysis

The mtDNA sequencing in the patient’s blood revealed a heteroplasmic variant in *MT-ND1,* NC_012920.1:m.4135T>C (p.Tyr277His). Using MitImpact (Castellana et al., 2021), 11 out of 16 pathogenicity predictors evaluate the variant as pathogenic, but according to the Apogee score (0.38), the variant is likely benign (Castellana et al., 2017). The presence of the variant was confirmed in other tissues, and heteroplasmy levels were determined (93% in muscle, 92% in buccal swabs, 90% in blood and urinary epithelial cells, and 89% in fibroblasts). The variant NC_012920.1:m.4135T>C was found in the blood of the patient’s older brother at the heteroplasmy level of only 24%. Unfortunately, no samples from the patient’s mother were available for variant testing.

Using ACMG/AMP criteria adapted for mitochondrial variants (McCormick et al., 2020), the m.4135T>C variant was curated manually. The initial *in silico* analysis using the APOGEE algorithm (Castellana et al., 2017) resulted in a neutral or possibly benign impact of the variant; thus, BP4 criteria were applied. However, the variant demonstrated its damaging effect on CI in muscle and fibroblast mitochondria, which meets the PS3 criteria. Since the mutation load is markedly lower in the blood of an older healthy brother than the heteroplasmy in the proband, the PP1 criteria (a variant heteroplasmy level segregates with the phenotype in the family) may be applied. Similarly, the phenotype suggests single gene etiology (PP4 criteria). The allele frequency is 0.032%–0.046% in public databases [gnomAD (Laricchia et al., 2022)]; Helix (Bolze et al., 2019) and Mitomap [update 2023-3-1 (Lott et al., 2013)]. Therefore, PM2 or BA1 criteria cannot be applied. Taken together, the variant is classified as likely pathogenic based on meeting the PS3, PP1, PP4, and BP4 criteria.

The initial filtering of patient exome data resulted in a set of 976 variants (approximately half of them in known disease genes) that were not present in control databases in a homozygous state. No candidate variant(s) were prioritized by exome data analysis. Similarly, no candidate variant was found in CI-related or optic atrophy-related genes.

### 4.2 Structural characterization of the variant

**FIGURE 2:**
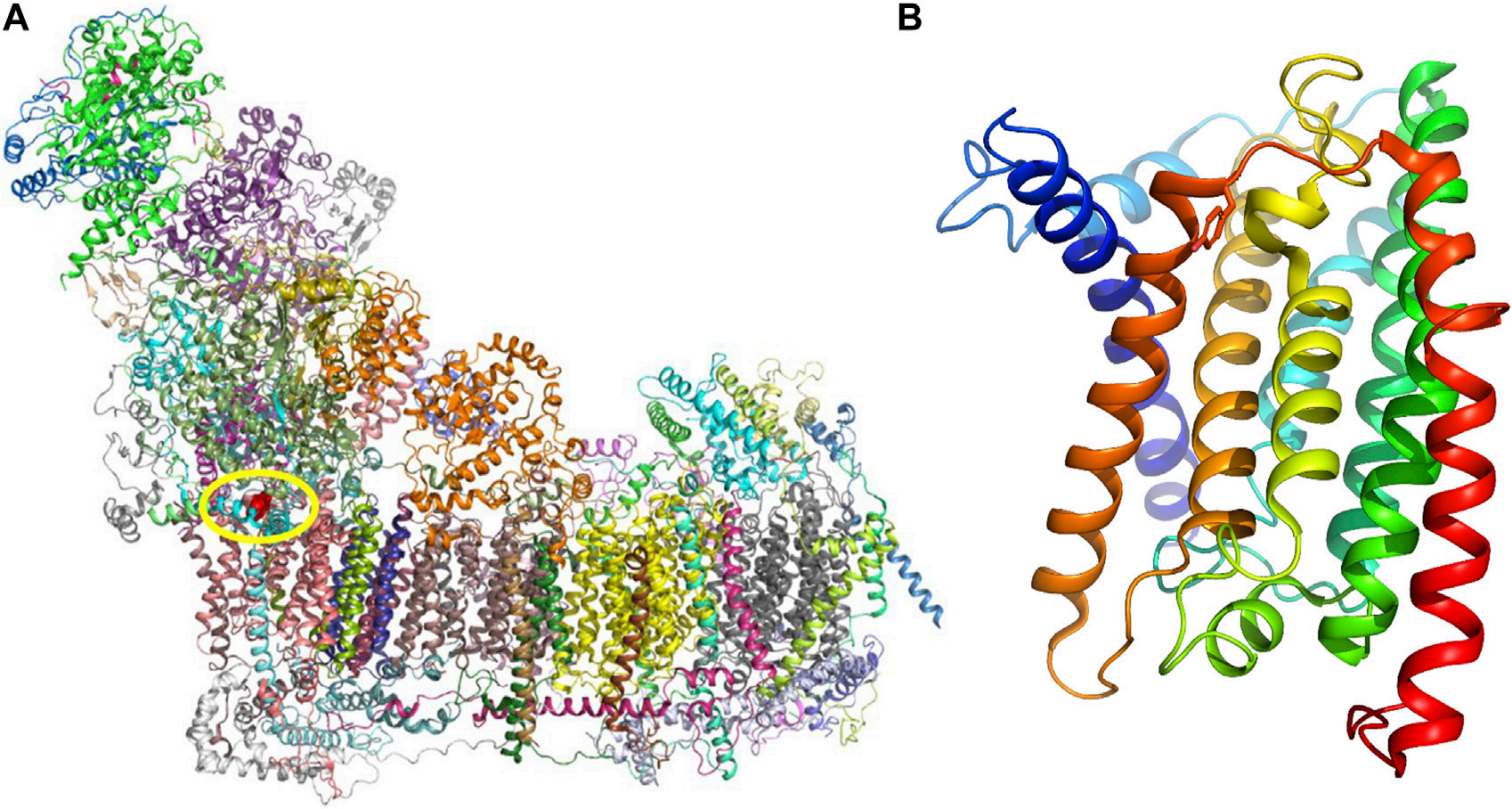
**(A)** Respiratory chain complex I. Individual protein molecules in the cartoon representation are shown in various colors. Subunit ND1 is shown in pink, and tyrosine residue 277 (Tyr277) atoms are rendered red spheres**. (B)** Structure of the ND1 subunit is composed of eight transmembrane helices (TMHs), and helices in the cartoon representation are shown in rainbow colors (blue in the N-terminus and red in the C-terminus). Tyr277 is located at the end of TMH7 (dark orange).

The affected ND1 subunit (NADH-ubiquinone oxidoreductase chain 1) forms a part of the membrane portion of the CI (Figure 2A). Human ND1 has eight transmembrane helices (TMH), and Tyr277 is located at the matrix end of TMH7 (Figure 2B). Tyr277 lies on the triple border of subunits ND1, NDUFS2, and NDUFS8, forming the mouth of a hydrophobic channel that connects the matrix with the inner hydrophobic membrane segment. The side chain of Tyr277 is hydrogen-bonded with Leu64 of the NDUFS8 subunit. The closest NDUFS2 subunit residue–Asn265 is 3.7 Å away, making only a weak vdW contact. The channel is in the cryo-EM structure (PDB:5XTD) occupied by a phospholipid molecule (Supplementary Figure S1). In addition, we noticed that the reported variant site on TMH7 is not far from the ubiquinone access channel. The closest interatomic distance between Tyr277 and ubiquinone in the bovine active form is 11.3 Å. This distance is not sufficient to alter, e.g., the shape of the ubiquinone binding cavity, but it does not exclude the possible alteration of long-range electrostatic interactions and electron transfer path. However, it is difficult to speculate on this as ubiquinone reduction and energy transduction mechanisms are still insufficiently understood (Chung et al., 2022).

Tyr277 is highly conserved in ND1 subunits across mammals. However, in the larger kingdom of organisms (including prokaryotes), its position is also frequently occupied by other hydrophobic residues, e.g., Leu (Supplementary Figure S2). The gene variant reported here results in substituting Tyr residue with a smaller and more polar His residue.

**FIGURE 3:**
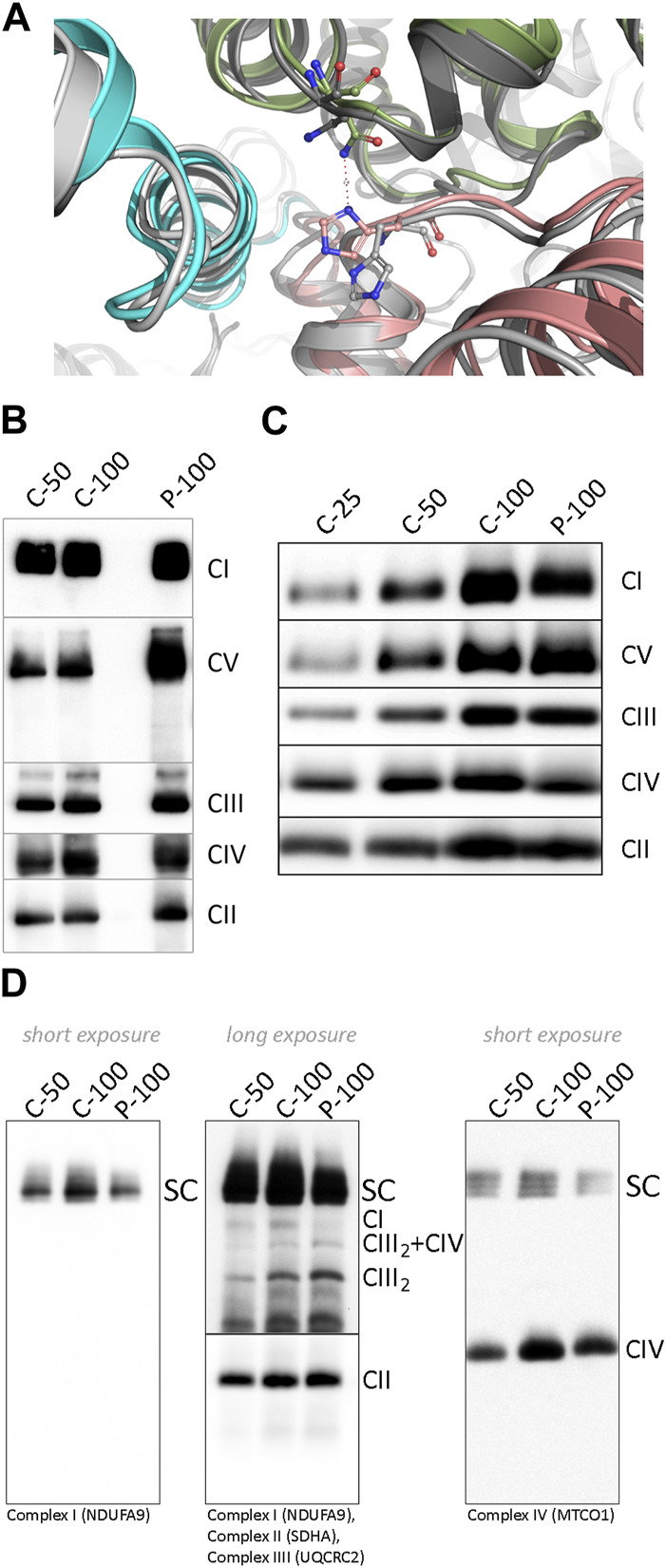
**(A)** Difference in the histidine position and its interactions in two ND1 Tyr277His - upper index mutant forms. The mutated active CI form is shown in greyscale and its inactive state in colors. His277 of subunit ND1 (pink) is in the inactive form flipped upward toward subunit NDUFS2 (green), where it forms an H-bond with the side chain of Asn232, and the NDUFS8 subunit is shown in blue. **(B)** BN-PAGE analysis of dodecylmaltoside-solubilized mitochondria from control and patient’s muscle and **(C)** cultivated skin fibroblasts. **(D)** BN-PAGE/WB analysis of digitonin-solubilized mitochondria from control and patient’s fibroblast. The numbers 25, 50, and 100 show the loading doses of the protein. Relative signal intensity was normalized to the intensity of complex II by densitometric analysis. Abbreviations: C, control; CI–CV, complex I-V; P, patient; SC, supercomplexes (I+III_2_, I+III_2_+IV, I+III_2_+IV_2_); CIII_2_+IV, supercomplex containing CIII-dimer and CIV; CIII_2_, CIII-dimer.

To predict the effect of Tyr277 substitution by His on the ND1 protein structure and its function within CI, we used DynaMut software (Rodrigues et al., 2018). Tyr277His mutation is predicted to destabilize the metabolically active state of CI. On the other hand, the inactive state that does not support respiration activity is predicted to be slightly stabilized (Supplementary Table S1). The introduced His residue is shorter than Tyr; therefore, it cannot form the hydrogen bond with Leu64 of the NDUFS8 subunit (Supplementary Figure S1), thus destabilizing both the active and inactive CI forms. The stabilizing effect of mutation on the inactive CI form could be explained by forming the new inter-subunit H-bond between the imidazole ring of His277 (subunit ND1) and Asn232 (equivalent to the Asn265 in human) sidechain of the subunit NDUFS2 (Figure 3A). This H-bond was not found in the active form probably due to the unfavorable position of the Asn residue. The predicted energy difference between the active and inactive states indicates that the population ratio between corresponding CI forms is altered by more than 50% (favoring the inactive state), thus significantly decreasing respiration efficiency. We also wanted to confirm these results using the recently published cryo-EM structures of bovine CI (Chung et al., 2022). Again, the active bovine CI with bound ubiquinone was predicted to be destabilized, while the inactive complex without ubiquinone was stabilized (Supplementary Table S1), thus predicting more than 32% drop in the respiration rate.

### 4.3 Functional characterization

To characterize the impact of the NC_012920.1:m.4135T>C variant on the structure and function of the OXPHOS system, we analyzed steady-state levels of OXPHOS complexes in isolated mitochondria from muscle and fibroblasts (Figures 3B–D). The amount of CI holoenzyme was decreased to approximately 80% in muscle and 90% in fibroblasts, and no accumulation of CI assembly intermediates was found. The amount of CIV was reduced below 80% of control in both tissues, and the amount of ATP synthase was elevated. Analysis of DIG-solubilized mitochondria revealed a reduced amount of CI-containing SCs and an increased level of CIII-dimer in fibroblasts. Due to the limited amount of the obtained tissue, analysis of SCs from muscle was not performed.

Although the amount of CI in muscle mitochondria was decreased only to approximately 80–90%, its activity decreased to approximately 60% and CI + III to 80% of the lower limit of control values (Table 1). Reduced activities of CI and CI + III led to significantly increased CII, CII + III, CIII, and CIV activities, suggesting a compensatory effect (Table 1) in the muscle. Histochemistry in the skeletal muscle biopsy revealed focal subsarcolemmal accumulation of the SDH reaction product in approximately 5% of muscle fibers. In fibroblasts, the CI + III activity was below the lower limit of control.

**TABLE 1:** Activities of respiratory chain complexes (nmol∙min^-1^∙mg^-1^ protein) in isolated mitochondria from muscle and fibroblasts.

	Isolated muscle mitochondria		Fibroblasts	
	Patient	Age-related controls (*n* = 30)	Patient	Age-related controls (*n* = 15)
CI	63	110–290	32	12–66
CI + III	102	126–316	9	10–30
CII	312	21–93	14	5–29
CII + III	532	82–251	NA	NA
CIII	935	>200	NA	NA
CIV	2,673	658–1,552	14	5–26
CS	1,395	435–1,234	24	13–46

Abbreviations: CI: complex I, NADH, coenzyme Q reductase; CI + III: complex I + III, NADH, cytochrome *c* reductase; CII: complex II, succinate coenzyme Q reductase; CII + III: complex II + III, succinate cytochrome *c* reductase; CIII: complex III, coenzyme Q cytochrome *c* oxidoreductase; CIV: complex IV, cytochrome *c* oxidase; CS: citrate synthase; n: number of controls; NA: not analyzed.

## 5 Discussion

The patient’s clinical manifestation was somewhat unique compared to a classic LHON manifestation. He suffered from a typical bilateral painless decrease in visual functions. However, the BCVA at the nadir did not decline to a level observed in LHON patients carrying one of the three prevalent pathogenic variants (Chun and Rizzo, 2017; Newman et al., 2020). The patient was referred to our center at the later stage of the disease, so we do not know if an acute phase was present during the disease onset. The presence of Uhthoff’s phenomenon is clinically interesting, which is relatively rare in LHON (Riordan-Eva et al., 1995).

Both homoplasmic and heteroplasmic pathogenic variants of *MT-ND* genes have been associated with LHON or LHON-like phenotype. Over 90% of LHON patients are carriers of one of the most frequent pathogenic variants: m.11778G>A, m.14484T>C, or m.3460G>A (Carelli et al., 2004). These variants are necessary but insufficient to cause blindness, and other environmental factors [e.g., smoking (Kirkman et al., 2009)] are needed to trigger retinal ganglion cell degeneration. Although the variant m.4135T>C was present with 0.042% (the MITOMAP database (Lott et al., 2013)), similar to the frequency of the variant m.11778G>A, our data provide compelling evidence supporting the causal role of the variant m.4135T>C in the patient’s phenotype. In the family, the m.4135T>C heteroplasmy level segregates with the phenotype. The mutation load of the variant was markedly higher in the patient’s tissues (89–93%) compared to the heteroplasmy level found in the blood (24%) of his healthy older brother, who is also a smoker.

The heteroplasmic m.4135T>C variant only leads to a slight decrease in CI, but the formation of SCs and enzyme activity are disturbed significantly. The disrupted formation of SCs leads to increased CIII-dimer, also reported in cybrids carrying a homoplasmic variant in *MT-ND1* (Lim et al., 2016) or mouse fibroblasts in *MT-ND2* variants (Marco-Brualla et al., 2019). Although CIII-dimer remains stable in the absence of the formation of CI-containing SC, the steady-state level of CIV decreased probably due to reduced stability in the absence of SCs formation (Lim et al., 2016). Recently, analysis of cybrid cells with m.3955G>A showed a significantly reduced CI holoenzyme and CI-containing SC (detected by NDUFS2 antibody). Nevertheless, UQCRC2 or COXIV detections showed similar signals across wild-type and mutant cells (Xu et al., 2022).

We hypothesized that Tyr277His mutation in the ND1 subunit alters the CI 3D structure, thereby disrupting the formation of SCs. The theoretical predictions also propose that predominantly substituting hydrophobic Tyr to hydrophilic His at position 277 could alter the natural equilibrium between active and inactive forms of CI, e.g., by forming a new inter-subunit H-bond between subunits ND1 and NDUFS2 in the inactive form, thus altering the allosteric regulation of the respiration chain activity. The resulting drop in the population ratio between the active and inactive CI forms might significantly contribute to the decreased function efficiency.

The measured activities of respiratory chain complexes presented here show that ubiquinone reduction within the patient samples still occurs since the CI + III activity is only partially disturbed, but due to decreased ability to form SCs, cells preferred alternative electrons flow through CII (elevated CII and CII + III activities) and other pathways. Second, due to the stabilizing effect of the variant on the inactive form of CI, where ubiquinone does not bind to the ubiquinone binding cavity of CI (Kampjut and Sazanov, 2020), CI-containing SCs are not assembled. Instead, other pathways with a source of electrons for ubiquinone are boosted, and the activities of the remaining respiratory chain complexes are elevated.

In conclusion, we have shown that although the CI is successfully assembled, the activity of CI and CI + III in muscle and fibroblasts and the ability to form SCs is decreased in patient fibroblasts with the m.4135T>C variant. The decreased respiratory efficiency of the mutated protein complex is probably due to a barrier in the transition from the inactive to the active state. We speculate that a combination of external factors (e.g., smoking and muscle exertion) was necessary for the patient’s phenotype manifestation, which is milder than usually observed in LHON patients.

## Data Availability

The datasets for this article are not publicly available due to concerns regarding participant/patient anonymity. Requests to access the datasets should be directed to the corresponding author.
