# Supplementary material for: Case report: A rare variant m.4135T>C in the MT-ND1 gene leads to Leber hereditary optic neuropathy and altered respiratory chain supercomplexes
Source: Front Genet. 2023 May 18;14:1182288. doi: 10.3389/fgene.2023.1182288 (PMC10233053; doi:10.3389/fgene.2023.1182288)
Supplement: Supplementary file 1 [file DataSheet2.pdf]

Supplementary Material

Case report: A rare variant m.4135T>C in the *MT-ND1* gene leads to Leber hereditary optic neuropathy and altered respiratory chain supercomplexes

Tereza Rákosníková[1], Silvie Kelifová[1], Hana Štufková[1], Kateřina Lokvencová[1], Petra Lišková[2], Bohdan Kousal[2], Tomáš Honzík[1], Hana Hansíková[1], Václav Martínek[3], Markéta Tesařová[1]*

*** Correspondence:** Markéta Tesařová: marketa.tesarova@lf1.cuni.cz

1 Supplementary Figures and Tables

1.1 Supplementary Figures

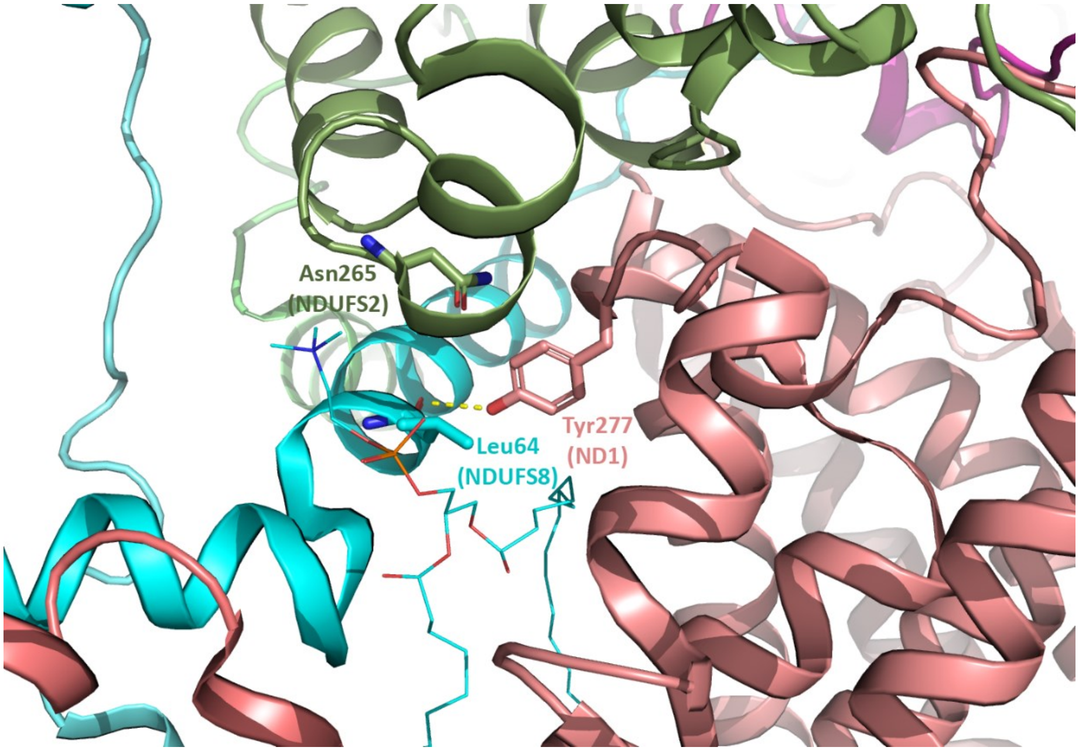

Supplementary Figure S1: Detail of the mouth of a hydrophobic channel that connects the matrix with the inner hydrophobic membrane segment. The side chain of tyrosine 277 of the ND1 subunit interacts with leucine 64 of the NDUFS8 subunit. The phospholipid molecule, occupying the channel in the PDB:5XTD is depicted together with asparagine 265 of the NDUFS2 subunit.

human ND1 sequence coloured by conservancy

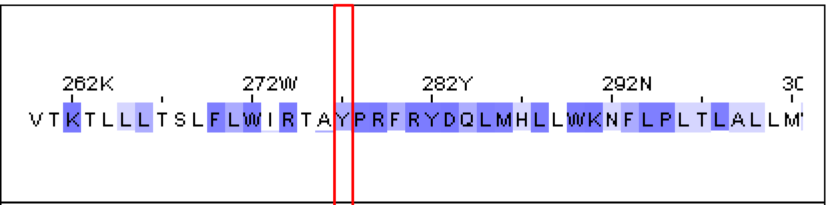

Conservation

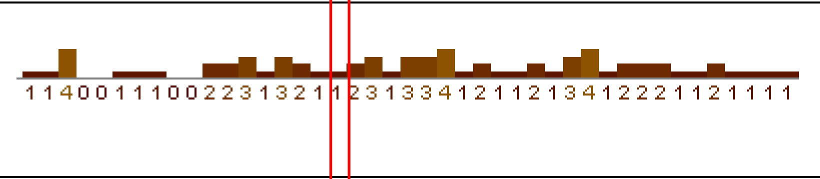

Consensus

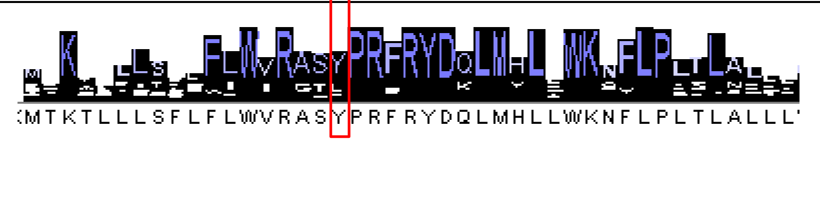

Supplementary Figure S2: Conservation of the human ND1 protein. Position 277 is conserved for tyrosine across mammals, but across a large group of organisms (including prokaryotes), position 277 is semi-conserved, meaning that position is conserved for hydrophobic residue.

1.2 Supplementary Table

Supplementary Table S1: DynaMut prediction outcomes. Structures of human CI in both active and deactive states are not available yet. However, we can assume that the effect of the mutation could be very similar to the animal models, as they show high homology with human ND1 orthologue (mouse 90% and bovine 87%); therefore, also Tyr277His variant in human ND1 likely decreases the CI efficiency by increasing concentration of deactive form on the expense of the active CI form.

mutation	prediction outcome ΔΔG [kcal/mol]	complex I	organism	PDB	reference
Tyr277His	− 0.226 (destabilising)	active state	Mus musculus	6G2J	(Agip et al., 2018)
Tyr277His	0.046 (stabilising)	deactive state	Mus musculus	6G72	(Agip et al., 2018)
Tyr277His	− 0.103 kcal/mol (destabilising)	active state	Bos taurus	7QSK	(Chung et al., 2022)
Tyr277His	0.06 kcal/mol (stabilising)	deactive state	Bos taurus	7QSN	(Chung et al., 2022)

2 References

Agip, A.-N. A., Blaza, J. N., Bridges, H. R., Viscomi, C., Rawson, S., Muench, S. P., et al. (2018). Cryo-EM structures of complex I from mouse heart mitochondria in two biochemically defined states. *Nat Struct Mol Biol* 25, 548–556. doi: 10.1038/s41594-018-0073-1.

Chung, I., Wright, J. J., Bridges, H. R., Ivanov, B. S., Biner, O., Pereira, C. S., et al. (2022). Cryo-EM structures define ubiquinone-10 binding to mitochondrial complex I and conformational transitions accompanying Q-site occupancy. *Nat Commun* 13, 2758. doi: 10.1038/s41467-022-30506-1.
